# Supplementary material for: Proteomic Analysis of Retinal Mitochondria-Associated ER Membranes Identified Novel Proteins of Retinal Degeneration in Long-Term Diabetes
Source: Cells. 2022 Sep 9;11(18):2819. doi: 10.3390/cells11182819 (PMC9497316; doi:10.3390/cells11182819)
Supplement: Supplementary file 1 [file cells-11-02819-s001.zip › cells-1751401 Supplemental Figure S1.pdf]

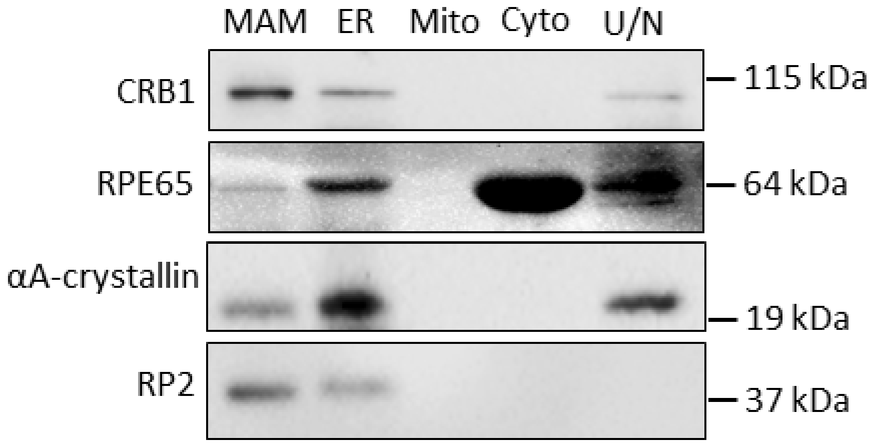

Supplemental Figure S1. Protein lysate of MAM and other subcellular fractions isolated from rat retinas were loaded into a SDS-PAGE system. Membrane was incubated with the following primary antibodies overnight at 4 degree: anti-CRB1 antibody (Boster Bio, A01499-1), anti-RPE65 antibody (Abcam, ab13826), anti-αA-crystallin antibody (Santa Cruz Technology, sc-28306), anti-RP2 antibody (Santa Cruz Technology, sc-390220). After incubation with HRP-conjugated secondary antibodies, membranes were developed with Clarity and Clarity Max ECL Western Blotting Substrate (Bio-Rad, CA, USA) using Chemi-Doc MP Imaging System (Bio-Rad, CA, USA). MAM: Mitochondria-associated ER membrane; ER: endoplasmic reticulum; Mito: mitochondria; Cyto: cytoplasm; U/N: unbroken cells with nuclei; CRB1: Crumbs Cell Polarity Complex Component 1; RPE65: Retinal Pigment Epithelium-specific 65 kDa; RP2: Retinitis Pigmentosa 2.
